# Coproporphyrin III Produced by the Bacterium *Glutamicibacter arilaitensis* Binds Zinc and Is Upregulated by Fungi in Cheese Rinds

**DOI:** 10.1128/mSystems.00036-18

**Published:** 2018-08-21

**Authors:** Jessica L. Cleary, Shilpa Kolachina, Benjamin E. Wolfe, Laura M. Sanchez

**Affiliations:** a Department of Medicinal Chemistry and Pharmacognosy, University of Illinois at Chicago, Chicago, Illinois, USA; b Department of Biology, Tufts University, Medford, Massachusetts, USA; Northwestern University

**Keywords:** Glutamicibacter arilaitensis, imaging mass spectrometry, natural rind cheese, *Penicillium*, specialized metabolites, zinc coproporphyrin III

## Abstract

Microbial communities of fermented food microbiomes typically exhibit predictable patterns of microbial succession. However, the biochemical mechanisms that control the diversity and dynamics of these communities are not well described. Interactions between bacteria and fungi may be one mechanism controlling the development of cheese rind microbiomes. This study characterizes a specific bacterium-fungus interaction previously discovered on cheese rinds between the bacterium Glutamicibacter arilaitensis (formerly Arthrobacter arilaitensis) and fungi of the genus *Penicillium* and identifies the specialized metabolites produced during cocultures. G. arilaitensis was previously shown to produce an unknown pink pigment in response to the presence of *Penicillium*. Using a combination of mass spectrometry, nuclear magnetic resonance (NMR), and transcriptome sequencing (RNA-seq), we determined that this pigment production is associated with production of coproporphyrin III. The discovery that coproporphyrin III preferentially bound zinc over other trace metals found in cheese curds highlights the value of using analytical chemistry to confirm identity of predicted chemical species.

**IMPORTANCE** Bacterium-fungus interactions play key roles in the assembly of cheese rind microbial communities, but the molecular mechanisms underlying these interactions are poorly characterized. Moreover, millions of people around the world enjoy eating cheeses and cheese rinds, but our understanding of the diversity of microbial metabolites ingested during cheese consumption is limited. The discovery of zinc coproporphyrin III as the cause of pink pigment production by Glutamicibacter arilaitensis suggests that secretion of this molecule is important for microbial acquisition of trace metals.

**Author Video**: An author video summary of this article is available.

## Abstract (precis)

Bacterium-fungus interactions play key roles in the assembly of cheese rind microbial communities, but the molecular mechanisms underlying these interactions are poorly characterized. Moreover, millions of people around the world enjoy eating cheeses and cheese rinds, but our understanding of the diversity of microbial metabolites ingested during cheese consumption is limited. The discovery of zinc coproporphyrin III as the cause of pink pigment production by Glutamicibacter arilaitensis suggests that secretion of this molecule is important for microbial acquisition of trace metals.

## INTRODUCTION

Cheese rinds form on the surfaces of naturally aged cheeses and provide distinct esthetics and sensory qualities. Despite the widespread consumption of these economically and culturally significant microbial communities, surprisingly little is known about the mechanisms driving the assembly of cheese rinds. Recent studies using high-throughput sequencing approaches have mapped out the diversity of bacteria and fungi in cheese rinds from cheeses in Europe and North America (1–4). While the community composition of rinds varies across different styles of cheeses, reproducible patterns of community development have been documented across batches of cheeses (4, 5). The molecular and genetic mechanisms driving these reproducible patterns of succession are poorly characterized. The relatively low complexity of cheese rind communities and ability to reconstruct rind microbial communities *in vitro* provide opportunities to link genetic and molecular mechanisms with community-level processes.

One potential process controlling cheese rind community development could be interactions between bacteria and fungi. Previous work from our labs and others has demonstrated strong bacterium-fungus interactions in cheese rinds (5–8). In a screen of the most abundant cheese rind bacteria and fungi, we found that bacteria were highly responsive to fungi as measured by growth responses (change in CFU) while fungi had very weak responses to bacteria (4). Previous studies have focused on alteration of cheese pH as a mediator of cheese rind microbial interactions, but the genetic and molecular mechanisms underlying most interactions in cheese rinds have not been determined (4).

Competition for trace metals may be one mediator of microbe-microbe interactions in cheese rinds. Iron is known to limit the growth of cheese rind bacteria (9, 10), and our recent study of the ecological distributions of *Staphylococcus* species in cheese rinds found that iron may mediate interactions between fungi and *Staphylococcus* spp. (5). The finding that a high frequency of horizontally transferred genes is related to iron acquisition also suggests that iron acquisition is a key process in cheese rinds (11). Other trace metals are implicated in microbial growth as well, including copper, magnesium, cobalt, manganese, and zinc (12). A growing wealth of information also indicates that copper and zinc can play a role in antibiotic resistance either by physically enhancing or inhibiting antibiotic behavior or by providing selective pressure toward antibiotic resistance (13). However, the genetic and molecular mechanisms underlying metal acquisition and metabolism during microbe-microbe interactions on cheese rinds are poorly characterized.

**FIG 1:**
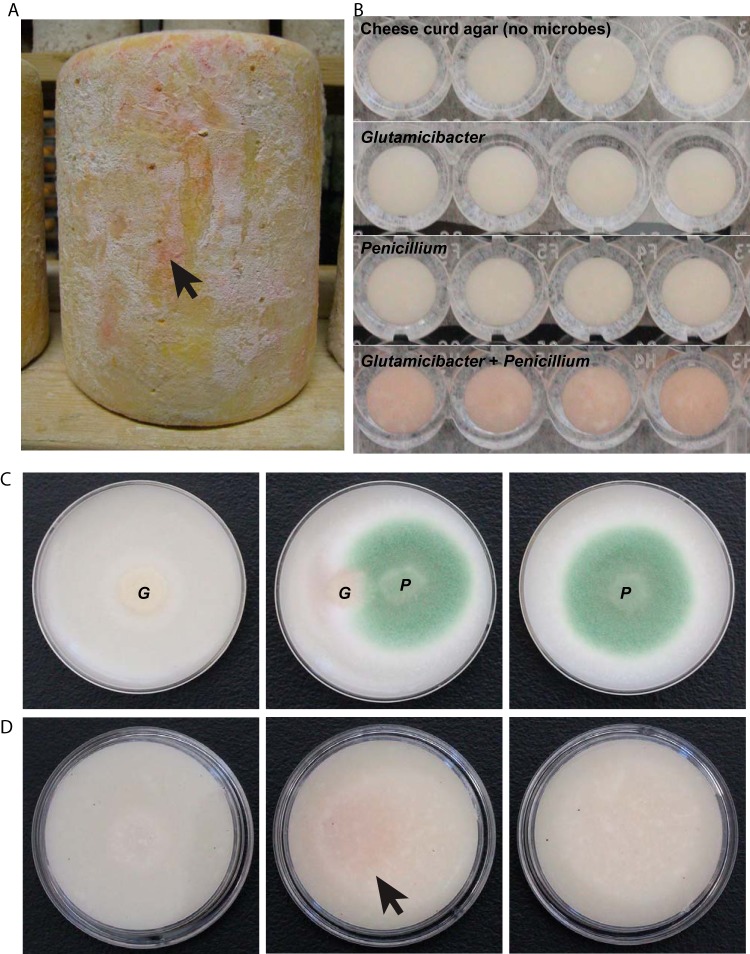
(A) Pink marbling on the surface of aged cheese rinds is sometimes observed during the cheesemaking process. It is likely that this pink pigment is produced by microbes growing on the rinds and has remained unidentified. (B) Observations in well plate assays showed that G. arilaitensis (G) produces a pink pigment when grown alongside *Penicillium* (P). (C and D) Colonies grown on agar plates are shown from top (C) and bottom (D) views. Photos were taken 5 days after inoculation of cheese curd agar.

This study investigated a specific interaction between two cheese microbes in order to elucidate mechanisms of metal acquisition in cheese rinds. In our previous work, when the bacterium Glutamicibacter arilaitensis (formerly known as Arthrobacter arilaitensis) was grown with filamentous fungi, it resulted in the striking phenotype of pink pigment secretion (Fig. 1) (4). We have observed that pigmentation can also occur when the bacterium is grown by itself but occurs much more rapidly when grown next to or cocultured with filamentous fungi (4). G. arilaitensis is widespread in surface-ripened cheeses and is commonly used as a starter culture in cheese production (4, 14, 15). G. arilaitensis has been reported to produce desirable yellow and red-brown pigments in surface-ripened cheeses (14, 16), but the genetic and molecular causes of pigment production by G. arilaitensis have not been previously characterized. Chemical characterization of the siderophores and other metal-chelating compounds in question is necessary to better define the biochemical basis of trace metal acquisition in cheese rinds (17).

In this work, we have identified coproporphyrin III as a metabolite produced by G. arilaitensis in response to *Penicillium* and exogenous siderophores, such as desferrioxamine B. Extracted and purified coproporphyrin III was found to be bound to zinc in excess over other metals, including iron, which suggests an important and previously unrecognized role of zinc in microbial interactions in cheese rinds. Matrix-assisted laser desorption ionization–time of flight imaging mass spectrometry (MALDI-TOF IMS) was used to detect changes in small-molecule production and biosynthesis in response to a *Penicillium* species commonly found on cheese rinds. This was confirmed by transcriptome sequencing (RNA-seq) which demonstrated that biosynthetic genes related to coproporphyrin production were also upregulated in the presence of *Penicillium*. Cheese curd supplemented with coproporphyrin III more strongly inhibits the growth of *Penicillium* than *Arthrobacter* and may help explain the previously described inhibitory effect of *Arthrobacter* on *Penicillium* (4). Production of a zinc-chelating compound suggests that zinc plays a key role in the growth of G. arilaitensis in cheese rinds.

## RESULTS

### Imaging intact bacterium-fungus interactions.

**FIG 2:**
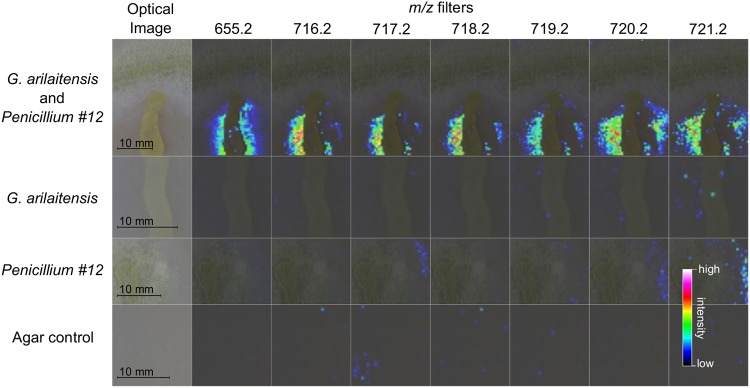
MALDI-TOF imaging mass spectrometry (IMS) for prioritization of *m/z* signals. Imaging performed on bacterial and fungal cultures allowed visualization of *m/z* distribution across the sample. Upregulation of *m/z* 655 was observed when *Penicillium* sp. 12 and G. arilaitensis were grown together on cheese curd agar plates and appears to be localized in bacterial colonies. Isotopes of zinc coproporphyrin III (716.2 through 721.2) also appear to be upregulated under coculture conditions.

Matrix-assisted laser desorption ionization–time of flight imaging mass spectrometry (MALDI-TOF IMS) was used to identify molecules produced when G. arilaitensis strain JB182 was grown in the presence of *Penicillium* sp. strain 12 (Fig. 2). The G. arilaitensis was isolated from a washed rind cheese made in Vermont. *Penicillium* sp. 12 was isolated from a natural rind cheese in the same cheese cave as the G. arilaitensis. This is an undescribed *Penicillium* species in section Viridicata and series Camemberti of the genus *Penicillium* and is a sister species to the clade of fungi that includes Penicillium camemberti, Penicillium fuscoglaucum, and Penicillium biforme (18). IMS allowed for the rapid visualization of the spatial distribution of molecules from bacteria and fungus grown on agar plates (19, 20). By looking for signals that increase in intensity when G. arilaitensis and *Penicillium* sp. 12 were grown in close proximity, we rapidly identified mass-to-charge (*m/z*) ratios of interest that could potentially represent compounds associated with this interaction. Retaining the spatial organization of microbial cultures also provides context for molecular production (i.e., excreted versus colony-associated molecules) that can be lost when extractions are performed (21).

Increased intensity of *m/z* 655 was observed only in bacterial colonies on coculture plates, suggesting that this particular molecule is produced by G. arilaitensis and upregulated in response to the *Penicillium*. Throughout the cheese aging process, the rind pH changes from pH 5 to pH 7 as microbial growth leads to deacidification of the top surface of the cheese curd (see Fig. S1 in the supplemental material) (5). Analyses were performed under both aged and unaged conditions to capture metabolites at different pH levels. At pH 5, we identified 10 *m/z* values at high intensity in bacterial colonies, 17 *m/z* values at high intensity in fungal colonies, and 2 *m/z* values at high intensity in coculture spots (co-spots) (Fig. S2). At pH 7, we identified 10 *m/z* values at high intensity in bacterial colonies, 22 *m/z* values at high intensity in fungal colonies, and 25 *m/z* values at high intensity in coculture spots (Fig. S3). Of all the *m/z* values, only two (*m/z* 655 and 623) were uniquely present in the coculture spots at both pH levels. The *m/z* 655 was prioritized for further chemical structure analysis because it appeared to be more localized to the bacterial colony and immediately surrounding agar, which would indicate that it was a bacterial metabolite and thus amenable to large-scale fermentation required for structure elucidation studies (Fig. 2).

10.1128/mSystems.00036-18.1 FIG S1  Optimizing microbial growth conditions. In order to place this interaction in environmental context, IMS was performed on cheese curd agar that was pH adjusted to 5 and 7. Attempts to alter salt content along with the pH showed that both G. arilaitensis and *Penicillium* sp. 12 do not grow as quickly and robustly under starting conditions as they do under aged cheese conditions. IMS with 10% salt was unsuccessful, as was decreasing to 8% salt, as the high-salt content prevents adequate adhesion of the agar to the steel MALDI plate. Thus, pH was the only variable that we were able to experimentally test via IMS. When medium was adjusted to pH 5 with 10% salt, bacteria and fungus did not grow well, and imaging on these spots was unsuccessful. Decreasing salt content to 8% was not sufficient for growth and imaging, so pH 5 with 3% salt was used for imaging purposes. This optimization process led to IMS being performed on both pH 5 and pH 7 with 3% salt. Download FIG S1, TIF file, 0.3 MB. Copyright © 2018 Cleary et al. 2018 Cleary et al. This content is distributed under the terms of the Creative Commons Attribution 4.0 International license.

10.1128/mSystems.00036-18.2 FIG S2  Imaging data for cultures grown at pH 5. All *m/z* signals found to have increased intensity only in certain spots compared with overall intensity were deemed of interest. Signals at *m/z* 623 and 655 are increased in co-spots of fungus and bacteria. *m/z* 655 corresponds to coproporphyrin as shown by extraction and further analysis. Extraction and analysis of pink pigment did not show *m/z* 623, which could be either a bacterial or a fungal metabolite. As evidenced by the presence of biosynthetic machinery in the G. arilaitensis genome for siderophores, 623.4 could represent desferrioxamine E listed in the GNPS library at *m/z* 623.34 [M + Na]. Download FIG S2, TIF file, 1.7 MB. Copyright © 2018 Cleary et al. 2018 Cleary et al. This content is distributed under the terms of the Creative Commons Attribution 4.0 International license.

10.1128/mSystems.00036-18.3 FIG S3  Imaging data for cultures grown at pH 7. As with Fig. S2, all *m/z* signals found to have increased intensity only in certain spots compared with overall intensity were deemed of interest. Many signals are likely due to isotopes (*m/z* 716 through 719) or analogues (*m/z* 515 and 531), but generally speaking, many fungal signals were found at lower mass ranges and bacterial signals were found in the higher ranges. *m/z* 623 and *m/z* 655 were again observed to be increased in interaction with the fungus. 655.8 is shown to demonstrate the ability of MALDI-TOF to distinguish between signal at 655.2 and molecules with similar weights. Download FIG S3, TIF file, 1.3 MB. Copyright © 2018 Cleary et al. 2018 Cleary et al. This content is distributed under the terms of the Creative Commons Attribution 4.0 International license.

### Culture of G. arilaitensis for large-scale pigment production.

In order to identify *m/z* 655 by an orthogonal liquid chromatography tandem mass spectrometry (LC-MS/MS) approach, large-scale liquid cultures of G. arilaitensis were grown in cheese curd medium. Filamentous fungi such as *Penicillium* do not readily grow in liquid culture; therefore, G. arilaitensis was grown alone and also treated with an exogenous siderophore, desferrioxamine B (Fig. S4A). We suspected iron competition to be the driving factor behind pigment production and therefore attempted to mimic *Penicillium* sp. 12 depletion of environmental iron by adding a metabolite (desferrioxamine B) closely resembling *Penicillium* iron-chelating molecules. Both isolated and treated cultures achieved pink pigmentation within 7 days of growth with differences in intensity and timing of appearance. Untreated cultures turned pink in 3 days and did not change in intensity over the course of 7 days, while treated cultures turned pink in 5 days with a deep red pigmentation by the end of 7 days.

10.1128/mSystems.00036-18.4 FIG S4  Photos of cultures of G. arilaitensis in liquid and solid cultures. (A) Liquid cultures of G. arilaitensis were both treated with desferrioxamine B and untreated. Cultures were grown in 10% cheese curd on a shaker at room temperature for 10 days before extraction. (B) Pigment production by Glutamicibacter arilaitensis JB182 in the presence of various *Penicillium* species. This experiment tested whether other species of *Penicillium* that grow on cheese can also cause upregulation of pink pigment production by Glutamicibacter arilaitensis JB182. At this time point, there is some pigment production from Glutamicibacter arilaitensis JB182 growing alone. Both Penicillium commune and Penicillium camemberti cause higher pigment production in a similar manner as *Penicillium* sp. 12. Penicillium nalgiovense produces penicillin and likely inhibits bacterial growth, which may explain the lack of pigmentation. Three replicate wells are shown. Both the top and bottom of the 96-well plate are shown. The image of the bottom of the plate was reflected in Adobe Illustrator to allow for the order of the wells of the top and bottom of the plate to be aligned. Download FIG S4, TIF file, 0.8 MB. Copyright © 2018 Cleary et al. 2018 Cleary et al. This content is distributed under the terms of the Creative Commons Attribution 4.0 International license.

### Culture of G. arilaitensis with additional *Penicillium* species.

To test whether pigment production is a conserved mechanism across cheese-derived *Penicillium* species, G. arilaitensis was grown in the presence of various *Penicillium* species. Similarly to liquid cultures supplemented with desferrioxamine, G. arilaitensis produces pink pigmentation in isolation, but an increase is observed when it is grown with *Penicillium* species (Fig. S4B). Both Penicillium commune and Penicillium camemberti cause higher pigment production in a similar manner as *Penicillium* sp. 12, suggesting that this is not just limited to *Penicillium* sp. 12. Penicillium nalgiovense produces penicillin and likely inhibits bacterial growth, which may explain the lack of pigmentation.

### LC-MS/MS and ICP-MS.

**FIG 3:**
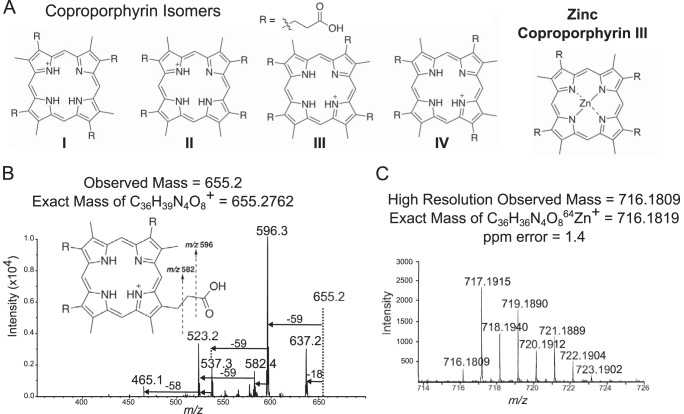
Mass spectrometry for identification of coproporphyrin. Tandem mass spectrometry strongly suggested a coproporphyrin, and high-resolution mass spectrometry matched zinc coproporphyrin. (A) Structural isomers of coproporphyrin are distinguished by the orientation of R groups around the porphyrin ring. (B) MS/MS fragmentation patterns of *m/z* 655 suggested a coproporphyrin identification but did not distinguish between structural isomers I to IV. Retention time matching was used to confirm identity of the coproporphyrin III isomer (Fig. 4). (C) High-resolution analysis of *m/z* 716 showed isotopic patterns consistent with that of zinc coproporphyrin III within acceptable parts-per-million error range.

Considering that mass spectrum (MS^1^) used for IMS on a MALDI-TOF spectrometer is not confirmatory of molecular identity, orthogonal techniques were used to conclusively identify the molecule with *m/z* 655. Both treated and untreated cultures were extracted for LC-MS analysis. LC-MS/MS was used to obtain fragmentation patterns of *m/z* 655. Tandem mass spectrometry data were dereplicated using Global Natural Products Social Molecular networking (GNPS), and bacterial extracts were matched with commercial standards that confirmed matching fragmentation patterns of the precursor ion 655 (Fig. 3) (22, 23). These data indicated that coproporphyrin was the main molecule responsible for the *m/z* 655 in the IMS experiment. ^1^H nuclear magnetic resonance (NMR) supports this molecular assignment (Fig. S5).

10.1128/mSystems.00036-18.5 FIG S5  NMR of purified G. arilaitensis extract. Proton NMR matches with reported NMR for zinc coproporphyrin III. Proton signals at 3.610, 10.035, 10.062, and 10.668 ppm are characteristic of the coproporphyrin III molecule. Download FIG S5, TIF file, 0.4 MB. Copyright © 2018 Cleary et al. 2018 Cleary et al. This content is distributed under the terms of the Creative Commons Attribution 4.0 International license.

**FIG 4:**
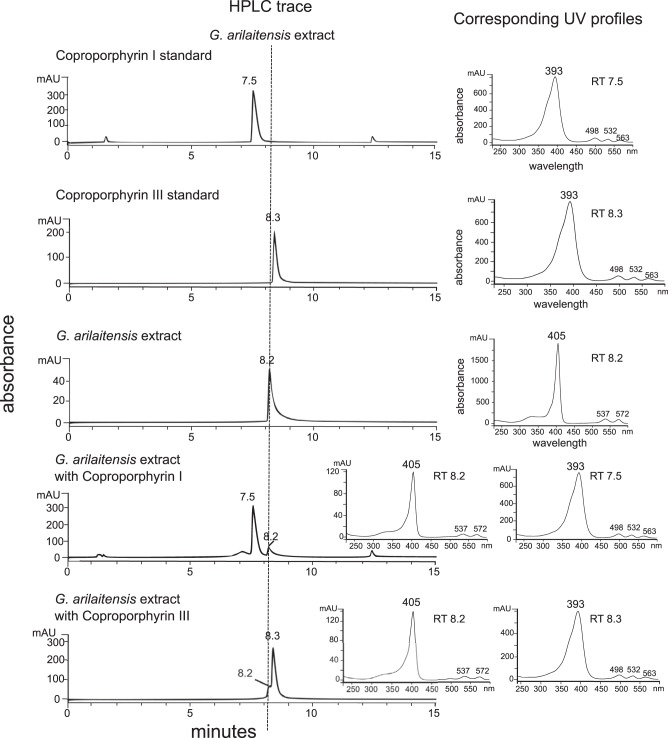
Retention times for identification of coproporphyrin. HPLC retention times (400 nm) confirm identity of zinc coproporphyrin III along with UV profiles. A gradient of 20% to 50% acetonitrile over 30 min gave retention times of standards and extract between 7 and 10 min. Extract did not match either standard despite the tandem mass spectrometry data indicating identity of coproporphyrin. This observation is consistent with zinc chelation, which has been reported to shift retention times to slightly more polar elution (27, 28). When standards were individually coinjected with the extract, the appearance of a small peak shoulder with coproporphyrin III that matched extract retention time indicated that the extract was in a chelated form. UV profiles also indicated metal chelation by extract as the Soret maximum was shifted slightly higher and smaller peaks correlated well with known zinc coproporphyrin III peaks (27, 28).

Fragmentation patterns of the precursor ion 655 also provided strong support for the identification as coproporphyrin (Fig. 3). Major fragments observed at *m/z* 637, 596, 537, and 523 correspond to fragmentation known for the coproporphyrins as seen in literature and in the GNPS database (24). Loss of an acetic acid via benzylic cleavage would result in the 596 fragment (−59 *m/z*), and loss of 2 acetic acids (depending on breakage point) would give rise to *m/z* 537 or 523 fragments (24). Although fragmentation patterns matched very well with standards, all constitutional isomers of coproporphyrin (I to IV) would have these same fragmentation patterns, so it is impossible to distinguish constitutional isomers based on MS/MS alone. Considering that isomers II and IV are rare forms, contributing 1% and 2% of all coproporphyrins, respectively (25, 26), the most likely candidates were I and III. Therefore, we used high-performance liquid chromatography (HPLC) retention time matching along with UV-visible (UV-vis) absorbance profiles to match to standards of I and III and confirmed identity of our isolated *m/z* 655 as coproporphyrin III (Fig. 4).

While working with different fractions of the G. arilaitensis extract, small shifts in retention time and peak broadening could not be explained by use of buffers, changes in injection volume, or column stationary phase. Accompanying changes in UV-vis profiles suggested chelation of metals by coproporphyrin (Fig. 4). As described by Mason et al., chelation of zinc by coproporphyrin shifts retention time to slightly more polar elution and exhibits UV profiles with a Soret maximum at 405 with additional α and β Soret maxima of a 1:1 ratio (27, 28). The peaks found at slightly more polar elution within the extract fit the description of zinc coproporphyrin III UV profiles, while the immediately following peaks fit the description of coproporphyrin III UV profiles (Fig. S6). Thus, the extract was found to contain both uncoordinated coproporphyrin III and zinc-bound coproporphyrin III, explaining the appearance of slight peak shoulders in coinjections.

10.1128/mSystems.00036-18.6 FIG S6  HPLC trace of G. arilaitensis extract with UV profiles. HPLC trace from gradient of 20 to 50% acetonitrile and 0.5 M ammonium acetate buffer over 30 min on a C_18_ column is shown here along with corresponding peak UV profiles. Retention times differ from Fig. 4 with the use of the buffer, and trace here is shown to demonstrate UV profiles. G. arilaitensis extract showed a large peak at retention time 7.4 of zinc coproporphyrin III (based on UV profile) as well as a small peak at retention time 7.6 of unchelated coproporphyrin III. Download FIG S6, TIF file, 0.1 MB. Copyright © 2018 Cleary et al. 2018 Cleary et al. This content is distributed under the terms of the Creative Commons Attribution 4.0 International license.

As soon as it became evident that the extract was chelating metals, the original MS analyses were revisited to look for possible *m/z* signals that could represent a chelated form of coproporphyrin III. When fractionated bacterial extract was submitted to HPLC for purification, all peaks at 400 nm were collected. Purified extract of coproporphyrin tended to display other ions at *m/z* 716, 717, 718, 719, and 720 in both LC-MS/MS and MALDI-TOF analyses (Fig. S7) that were resistant to robust separation from 655. Porphyrins are known to coordinate various metals aside from iron, and we hypothesized that these masses could correspond to copper or zinc addition to the coproporphyrin molecule.

10.1128/mSystems.00036-18.7 FIG S7  MS^1^ of purified G. arilaitensis extract. MALDI-TOF dried-droplet crystallization of G. arilaitensis extract purified by HPLC for 400-nm peaks was performed prior to elemental analysis. Coproporphyrin can readily be converted to methyl ester derivatives when dissolved in methanol under acidic conditions. Download FIG S7, TIF file, 0.3 MB. Copyright © 2018 Cleary et al. 2018 Cleary et al. This content is distributed under the terms of the Creative Commons Attribution 4.0 International license.

Fragmentation of *m/z* 716 suggested a zinc-coordinated coproporphyrin molecule as the fragmentation pattern of losses was similar to coproporphyrin III. The highest intensity at *m/z* 716 over other isotopes corresponds to the most abundant zinc isotope ^64^Zn forming an ion to show [M]^+·^ rather than [M+H]^+^. Elemental analysis showed that purified coproporphyrin bacterial extracts contained significant amounts of zinc (2.88 µg/mg of coproporphyrin [Fig. S8]), suggesting that coproporphyrin III has a higher affinity for zinc than other metals. Previous work has also reported that metal-containing porphyrins exhibit [M]^+·^ while coproporphyrins tend to become protonated (29). Furthermore, high-resolution analysis of the extract showed a mass of 716.1809, which is consistent with the formula C_36_H_36_N_4_O_8_^64^Zn^+^. The peak at 716.1809 was followed by a more intense peak at 717.1915 and six subsequent isotopologues at 1 Da apart (Fig. 3). To confirm that zinc was actually present and available for chelation in the cheese curd medium, a sample of cheese curd agar (CCA) was prepared in a similar fashion as for plating bacterial and fungal colonies and subjected to elemental analysis. The cheese curd agar was found to contain numerous trace metals, the most abundant of which was zinc (Fig. S8). Differing ratios of iron to zinc in the medium (about 1:3) compared to the purified coproporphyrin bacterial extract (about 1:25) suggest that although zinc was the most abundant trace metal present, the coproporphyrin III molecule still likely has a higher affinity for zinc than iron. This is especially supported by the presence of zinc-bound molecules in the IMS data (Fig. 2).

10.1128/mSystems.00036-18.8 FIG S8  Elemental analysis on G. arilaitensis extract and cheese curd agar. Elemental analysis performed via ICP-MS on G. arilaitensis extract purified by HPLC for 400-nm peaks (A) showed that zinc represents a disproportionately large amount of the sample. Boron, phosphorus, and calcium are likely contamination from the extraction, purification, and sample process, and the presence of iron and magnesium is not surprising as coproporphyrin is capable of chelating both metals. High levels of zinc compared with other trace elements were found in cheese curd agar medium (B). Higher ratios of zinc to iron in coproporphyrin III extract than in cheese curd medium suggest that coproporphyrin III has a higher affinity for zinc than iron (C). Download FIG S8, TIF file, 0.4 MB. Copyright © 2018 Cleary et al. 2018 Cleary et al. This content is distributed under the terms of the Creative Commons Attribution 4.0 International license.

Following other analyses that indicated the presence of zinc coproporphyrin III, reexamination of IMS data did show an upregulation of *m/z* 716, 717, 718, 719, and 720 on cheese curd agar (Fig. 2). On cheese curd agar mimicking unaged (starting) conditions (pH 5), high background at *m/z* 717 and *m/z* 718 from the agar made it difficult to distinguish upregulated signals, but *m/z* 716 could be interpreted to be increased surrounding bacterial colonies (Fig. S2). This could be explained by the observation that bacteria did not grow as well on pH 5 cheese curd agar as on pH 7 cheese curd agar (Fig. S1), resulting in lower excretion of bacterial metabolites.

### RNA-seq.

**FIG 5:**
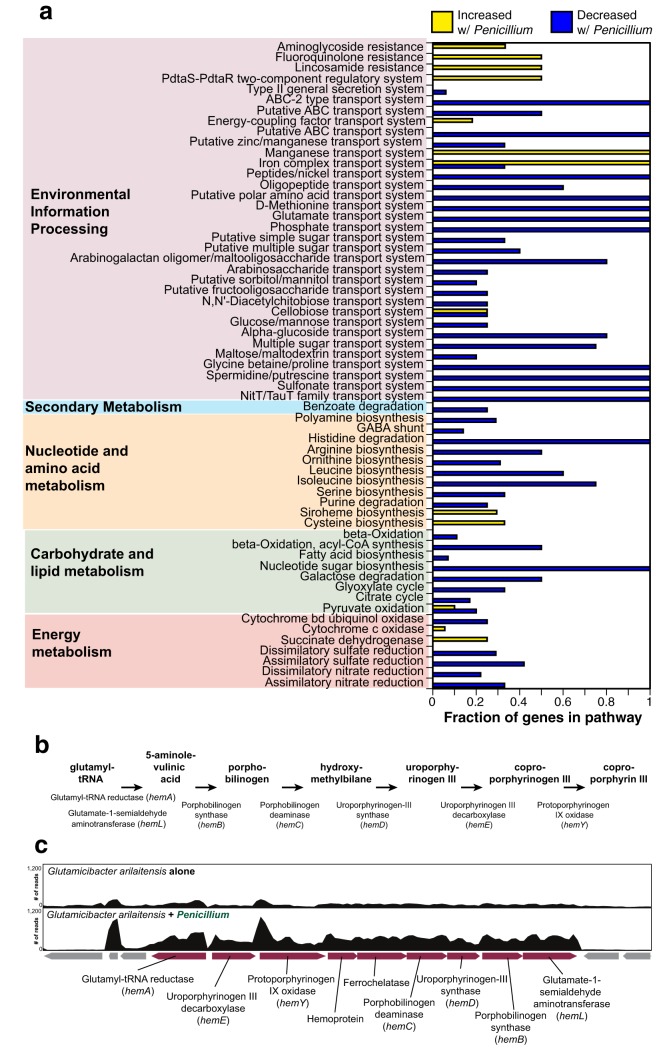
RNA-seq of Glutamicibacter arilaitensis alone and cocultured with *Penicillium* sp. 12. (a) Distribution of differentially expressed genes (greater than 3-fold change in expression) across KEGG pathways. Bars represent the proportions of components in the pathway that are represented in the differentially expressed genes. Yellow bars are genes that increased in expression with *Penicillium*, and blue bars are genes that decreased in expression with *Penicillium*. (b) A model of the coproporphyrin III pathway in *Actinobacteria*, based on reference 41. A detailed model is provided with molecular structures in Fig. S9 in the supplemental material. (c) Organization of the operon in the G. arilaitensis JB182 genome that contains homologs for genes in the coproporphyrin III pathway. The two panels above the operon show the coverage (number of reads mapped) for a representative transcriptome of G. arilaitensis JB182 growing alone (top) and G. arilaitensis JB182 cocultured with *Penicillium* sp. 12 (bottom).

To obtain additional support for a role of coproporphyrin in the interaction between G. arilaitensis and *Penicillium*, we performed RNA sequencing on G. arilaitensis grown alone and G. arilaitensis grown with *Penicillium* sp. 12 (Fig. 5). A very small subset of the data from this experiment for one siderophore operon has been previously published (11), but a thorough analysis of global expression data from the rest of the G. arilaitensis genome has not been previously presented.

10.1128/mSystems.00036-18.9 FIG S9  Chemical structures of the biosynthetic intermediates of the coproporphyrin III pathway. Download FIG S9, EPS file, 3.3 MB. Copyright © 2018 Cleary et al. 2018 Cleary et al. This content is distributed under the terms of the Creative Commons Attribution 4.0 International license.

Coculture of G. arilaitensis with *Penicillium* sp. 12 caused substantial transcriptional changes compared to growth of G. arilaitensis alone. Using a cutoff of 3-fold change in expression, 444 genes decreased in expression and 276 genes increased in expression (Table S1). Differentially expressed genes spanned a variety of functions as determined by KEGG pathway mapping via BlastKOALA (Fig. 5a). Many downregulated genes were associated with transport systems, with full pathways represented for the following transport systems: ABC and ABC-2 transporters, peptide/nickel, polar amino acids, d-methionine, glutamate, phosphate, glycine betaine/proline, spermidine/putrescine, and sulfonate. Amino acid biosynthesis pathways were also strongly downregulated with complete pathways represented by the differentially expressed genes for histidine degradation and partial pathways for arginine, leucine, and isoleucine biosynthesis. This collective downregulation of pathways associated with nutrient uptake and biosynthesis aligns with previous bacterium-fungus RNA-seq experiments from the cheese rind system demonstrating that fungi can dramatically increase nutrient availability through proteolysis of cheese curd (5).

10.1128/mSystems.00036-18.10 TABLE S1  RNA-seq results. Download TABLE S1, XLSX file, 0.1 MB. Copyright © 2018 Cleary et al. 2018 Cleary et al. This content is distributed under the terms of the Creative Commons Attribution 4.0 International license.

Full pathways that are represented by genes that increased in expression include those involved with manganese and iron transport systems, suggesting increased competition for metals when cocultured with *Penicillium*. One unique KEGG pathway to emerge in the upregulated genes is siroheme biosynthesis, which includes genes associated with coproporphyrin biosynthesis. Coproporphyrin III is part of the porphyrin biosynthesis pathway in bacteria (30). One model for coproporphyrin III production starts with the intermediate 5-aminolevulinic acid, yields uroporphyrinogen III as an intermediate, and ends with the oxidation of coproporphyrinogen III to yield coproporphyrin III (Fig. 5b). In some Gram-positive bacteria, the genes that encode the proteins in the porphyrin biosynthesis pathway have been found in operons, including the *hemAXCDBL* operon for biosynthesis of uroporphyrinogen III from glutamyl-tRNA (31, 32) and the *hemEYH* operon (33). In the genome of G. arilaitensis, we identified an operon of eight predicted coding sequences (locus tag Ga0099663_101508 to Ga0099663_101516) that contain homologs to these previously characterized components of the coproporphyrin III biosynthesis pathway (Fig. 5b). In support of the role of coproporphyrin III production in the interaction of G. arilaitensis with *Penicillium*, we see significantly increased expression (3- to 5-fold) of this entire operon compared to when G. arilaitensis is grown alone (Fig. 5c; Table S1).

### Growth assays with purified coproporphyrin III.

**FIG 6:**
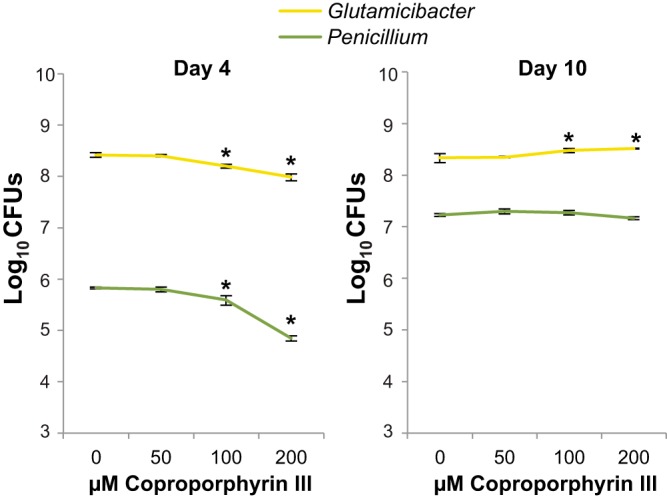
Growth of Glutamicibacter arilaitensis JB182 and *Penicillium* sp. 12 on cheese curd agar with a range of coproporphyrin III concentrations. Data represent means from four replicates, and error bars are standard deviations. Data were collected at 4 and 10 days after inoculation. Asterisks indicate treatments that are significantly different from 0 µM based on analysis of variance with Dunnett’s test (*P* < 0.05).

To test whether coproporphyrin III impacts the growth of G. arilaitensis or *Penicillium* sp. 12 growing on cheese curd, we supplemented cheese curd agar (pH 5) with purified coproporphyrin III dihydrochloride at a range of concentrations (0, 50, 100, and 200 µM). After 4 days of growth, both the 100 and 200 µM coproporphyrin III treatments had significantly lower growth for both G. arilaitensis (*F*_3,12_ = 69.8, *P* < 0.001) and *Penicillium* sp. 12 (*F*_3,12_ = 101.9, *P* < 0.001) (Fig. 6). Inhibition was much stronger for *Penicillium* at the 200 µM concentration (90% decrease at 200 µM) than for G. arilaitensis (63% decrease at 200 µM). At 10 days of growth, the inhibitory effect of coproporphyrin III was gone, with no significant differences in growth of *Penicillium* sp. 12 and slightly higher growth of G. arilaitensis in both the 100 and 200 µM coproporphyrin III treatments (Fig. 6).

## DISCUSSION

This study has discovered the chemical identity of a pigment produced during a potentially widespread bacterium-fungus interaction in cheese rinds. To our knowledge, this is the first time that coproporphyrin III has been definitively identified as a metabolite produced by G. arilaitensis and is the first identification of this compound from cheese microbes (34). The implications of production of this molecule are yet to be determined, but upregulation of production of coproporphyrin III by G. arilaitensis in the presence of the fungus *Penicillium* suggests that it plays a role in this specific bacterium-fungus interaction and possibly in rind microbial community development. The observed strong inhibition of *Penicillium* compared to G. arilaitensis at high concentrations suggests that coproporphyrin III has the potential to be part of the previously described inhibitory effect of G. arilaitensis on *Penicillium* (4). Coproporphyrin III may be produced by the bacterium during competition with the fungus for metals such as zinc and iron. Alternatively, the fungus may create a unique environment in the cheese, including altering the pH of the cheese, that enhances production of coproporphyrin III by G. arilaitensis. Considering that other cheese-derived *Penicillium* species can enhance pigment production by G. arilaitensis, this may be a conserved mechanism by which G. arilaitensis has evolved to exist on cheese rinds.

Other groups have characterized carotenoid pigments produced by G. arilaitensis, and other bacteria in the genus *Arthrobacter* (now *Glutamicibacter* for many species) have been shown to produce coproporphyrins (16, 30). The addition of manganese, zinc, and cobalt has also been shown to increase pigments due to coproporphyrin III production by Arthrobacter globiformis (31). These studies support the findings here but do not address the role that porphyrin production can play in interspecies interactions. In this case, coproporphyrin III has been shown to inhibit cell growth and is perhaps excreted in response to alterations in trace metal composition induced by the presence of *Penicillium* sp. 12. In light of past research showing that coproporphyrin III can mediate interactions between different bacterial species (32, 33), it would be interesting to explore if zinc coproporphyrin III production is found in other bacterium-fungus interactions outside cheese rind microbiomes.

Previous studies have investigated the impact of zinc supplementation in milk on lactic acid bacteria (LAB) (35) prior to the aging process and found that the presence of zinc alters the ability of LAB to acidify cheese during the initial cheesemaking process. This previous work was limited to LAB and did not include investigations of rind microbes, which include a wider variety of bacterial and fungal species. Addition of zinc feed additives has been reported to increase zinc levels in milk (36), and research on the elemental components of milk has shown differences in content due to variables related to seasonal changes (37). Considering the suggested variability of zinc that could be found in cheese, future studies by our group regarding coproporphyrin III production will include elemental analysis on all cheese curd batches.

The high levels of zinc compared to iron in the extracted coproporphyrin III suggest either that zinc chelation rather than iron chelation is the key task performed by this particular molecule, or as described by Anttila et al. (27, 28), that coproporphyrin III is a copper acquisition compound and zinc coproporphyrin III forms when copper is limited. Considering the high levels of zinc in comparison to other trace metals found in the elemental analysis of cheese curd medium, production of coproporphyrin III may be a mechanism by which G. arilaitensis acquires sufficient levels of zinc or avoids zinc toxicity. The results of elemental analysis of the cheese curd in this study were not surprising considering that zinc is primarily bound to casein, the primary component of cheese (35). However, there has long been documented an interplay between trace metal levels and porphyrin biosynthesis, and the results here suggest that trace metals are important considerations in microbial interactions (38). It would be interesting to determine if zinc coproporphyrin III production is necessary for successful cheese rind colonization or if it enhances the ability of G. arilaitensis to coexist in cheese rind microbial communities. Future research will also consider the potential for antimicrobial properties of zinc coproporphyrin III, as some iron chelators sometimes display antibacterial activity and zinc is known to enhance antibiotic effects (13, 39). The exact role of zinc-bound coproporphyrin III has not been elucidated here, and future efforts on our part will determine whether it is more responsive to fungal interaction or to abiotic factors.

The MALDI-TOF IMS method used here was crucial for rapid screening of potential *m/z* signal candidates responsible for pigmentation, as the spatial distribution of *m/z* peaks helps to determine the source when multiple species are present in a sample. Further culture, extraction, and confirmatory techniques are necessary to accomplish the goal of proving the identity of a particular molecule, but IMS allows minimally altered states of bacterial and fungal growth to be directly analyzed. In this case, the retrospective analysis of IMS data also showed *m/z* signals that were presumably due to zinc-chelated coproporphyrin III isotopes, which convincingly limits the possibility that the presence of zinc was an artifact of extraction and analytical techniques.

The additional unique signals observed in imaging have not yet been identified and represent a large amount of chemical information that is yet to be interpreted. It is likely that some of these metabolites play roles in community development and maintenance, and it is our goal to identify unknown signals shown in similar interactions between bacteria and fungi to build a foundation for molecular patterns and processes.

## MATERIALS AND METHODS

### General experimental procedures.

All HPLC analyses were performed on an Agilent 1260 Infinity equipped with a diode array detector and fraction collector (Agilent Technologies, Santa Clara, CA). Solvents used for the HPLC analysis were LC-MS grade and were used without further purification. Desferrioxamine standard was purchased from Sigma-Aldrich. Coproporphyrin I and III standards were purchased from Frontier Scientific. MALDI-TOF data were collected on a Bruker Autoflex Speed instrument. LC-MS/MS data were acquired using an HP1050 HPLC outfitted with a photodiode array (PDA) detector coupled to a Thermo Finnigan LCQ Advantage mass spectrometer. NMR spectra were acquired on a Bruker 400-MHz spectrometer equipped with a 5-mm PABBO z-gradient probe and referenced to residual solvent proton and carbon signals (δ_H_ 3.35, δ_C_ 49.3 for CD_3_OD), and LC-MS/MS analysis was performed on an Agilent 6300 ion trap. High-resolution MS^1^ data were collected on a Bruker Impact II quadrupole TOF (qTOF) instrument.

### Media and growth conditions.

G. arilaitensis JB182 was routinely grown on brain heart infusion (Bacto BHI) agar (BD) at room temperature (RT), and single colonies were selected for subsequent experiments. Liquid bacterial cultures were grown for 5 days at room temperature and 225 rpm in 4 ml of BHI medium. *Penicillium* sp. 12 was grown on plate count agar milk salt (PCAMS; 1 g/liter whole-milk powder, 1 g/liter dextrose, 2.5 g/liter yeast extract, 5 g/liter tryptone, 10 g/liter sodium chloride, 15 g/liter agar) plates at RT, and spores were harvested at 7 days (or until sporulation was observed) of growth for subsequent experiments.

### IMS.

G. arilaitensis was grown overnight in brain heart infusion (Bacto BHI) liquid medium (BD) at RT. Liquid cultures were normalized to an optical density (OD) of 0.1 and diluted 10^−2^ for working culture. Spores harvested from *Penicillium* sp. 12 were put into 1× phosphate-buffered saline (PBS), normalized to an OD of 0.1, and then diluted 10^−1^ for working culture. Five microliters of working cultures was spotted on thin (10 ml in 100-mm-diameter petri dish) 2.5% cheese curd agar (CCA; 25 g/liter lyophilized Bayley Hazen blue cheese curd, 5 g/liter xanthan gum, 30 g/liter NaCl, 17 g/liter agar, pH adjusted to 5.0 using NaOH). Thin agar plates were prepared for IMS by pouring approximately 10 ml (100-mm-diameter petri dish) of either 2.5% cheese curd agar with 3% NaCl adjusted to pH 5 using NaOH or 2.5% cheese curd agar with 3% NaCl adjusted to pH 7 using HCl.

After at least 5 days of growth, fungal spores were removed using a sterile Q-Tip with acetonitrile, and isolated bacteria, fungi, bacterium-fungus co-spots, and agar controls were excised from the agar plate and placed onto the steel MALDI plate. Pictures of wet agar spots on the steel plate were taken, and then MALDI matrix, 50:50 α-cyano-4-hydroxycinnamic acid (CHCA)–dihydroxybenzoic acid (DHB), was sieved (pH 5 cultures) or sprayed (pH 7 cultures) over the plate to completely cover all agar pieces with matrix. The plate was then put in the oven overnight at 30°C for drying. Once completely dry, excess matrix was removed from the plate, and after pictures were taken of the steel plate, it was inserted into the MALDI instrument. Using Bruker Flex Imaging v.4.1, the picture was matched to the steel plate inside the instrument and used to direct data acquisition to specific regions on the plate. Calibration was performed with Pepmix, and detection was set to 200 to 2,500 Da. Analysis was done in positive, reflectron mode with medium laser size and a frequency of 500 Hz. Five-hundred-micrometer raster points were chosen for all samples, and analysis was performed using Bruker Flex Imaging v. 4.1 with a total ion current (TIC) normalization applied.

### Extraction of biomolecules from liquid cultures.

G. arilaitensis was grown in small scale as described above. The resulting liquid culture was normalized to an OD at 600 nm (OD_600_) of 0.1 for inoculation into 50 ml of 10% liquid cheese curd medium (20 g/liter lyophilized Bayley Hazen blue cheese curd, 30 g/liter sodium chloride, autoclaved for 15 min).

Given that the interaction between G. arilaitensis and *Penicillium* appears to upregulate pigment production, two distinct culture conditions were used to measure and potentially upregulate production of *m/z* 655. Desferrioxamine B was sterile filtered through an 0.22-µm polyethersulfone (PES) membrane and added into two out of the four 10% liquid cheese curd medium flasks along with sterile glass beads, and all flasks were adjusted to a pH of 5. Flasks were autoclaved for 20 min (a short time is necessary to prevent burning of the curds) and after inoculation were incubated for 7 days at RT at 225 rpm. For large-scale cultures, 50-ml cultures were seeded into 1-liter flasks of 10% liquid cheese curd medium and desferrioxamine B was added (sterile filtered through 0.22-µm PES membrane).

Cultures were shaken at 225 rpm at RT for 10 days, and 20 g of Amberlite XAD16N resin was added to liquid cultures and shaken for 1 h. Cultures were then vacuum filtered to collect both resin and cells. This mixture, including the filter paper, was back-extracted with 50:50 dichloromethane (DCM)-methanol (MeOH) for 1 h at 225 rpm. The resulting slurry was vacuum filtered, and the filtrate was dried *in vacuo*. Crude biomass was subjected to solid-phase extraction (SPE) using a Supelco Discovery C_18_ cartridge (5 g; Bellefonte, PA). Elution occurred using a step gradient of 40 ml of MeOH–Milli-Q H_2_O solvent mixtures (10% MeOH, 20% MeOH, 40% MeOH, 60% MeOH, 80% MeOH, and 100% MeOH) and finally 100% ethyl acetate (EtOAc) to afford seven fractions. Each fraction was dried *in vacuo* and analyzed using MALDI-TOF MS. The 60% and 80% fractions were prioritized by MALDI-TOF as they contained the desired *m/z* 655 and were subjected to reverse-phase high-performance liquid chromatography (HPLC). Mass-guided HPLC isolation was conducted with a Kinetex C_18_ column (5 µm, 100 Å, 150 by 4.6 mm; Phenomenex, Torrance, CA), 75% MeOH-15% Milli-Q H_2_O (formic acid, 0.2% [vol/vol]), 1 ml/min, 210, 380, 400, and 600 nm. All peaks appearing at 400 nm were collected for MS analysis. These HPLC fractions were dried *in vacuo* and analyzed by ^1^H NMR. Retention time matching experiments were conducted with the same Kinetex C_18_ column with 0.5 M ammonium acetate buffer on a gradient of 20% to 50% acetonitrile over 30 min.

### Compound characterization.

Coproporphyrin III: red solid; ^1^H NMR [400 MHz, (CD_3_)_2_SO], 10.67 s, 10.06 s, 10.04 s, 8.48 s, 4.30 m, 3.61 bs, 3.58 s, 3.51 s, 3.35 bs HDO, 2.50 q (CD_3_)_2_SO, 2.16 t (*J* = 7.3 Hz), 1.87 s, 1.24 bs, 0.86 t (*J* = 6.5 Hz); high-resolution electrospray ionization mass spectrometry (HRESIMS) *m/z* [M]^+·^ 716.1809 (calculated for C_36_H_36_N_4_O_8_^64^Zn^+^, 716.1819).

### MS/MS analysis with GNPS.

The 400-nm peaks collected as described above were also subjected to MS/MS analysis for dereplication using the Global Natural Products Social Molecular Networking (gnps.ucsd.edu) database and workflow (22, 23). MS/MS analysis was done on a Thermo Finnigan LCQ Advantage Max mass spectrometer via direct infusion of purified G. arilaitensis extract. The electrospray ionization (ESI) conditions were set with the source voltage at 5 kV and capillary temperature at 250°C. The detection window was set from 200 to 2,000 Da, collision energy was at 50%, and isolation width was 3 *m/z*. High-resolution MS^1^ data were collected on a Bruker Impact II qTOF instrument in positive mode with the detection window set from 50 to 1,500 Da, on an ultra-HPLC (UHPLC) gradient of 10 to 100% acetonitrile over 10 min.

Tandem mass spectrum data were converted into mzXML files and submitted to the Global Natural Products Social Molecular Networking workflow in which fragmentation patterns are matched to library fragmentation data as well as other spectra within the same run (22, 23). This database identified coproporphyrin I and III as potential matches, with a cosine score of 0.93 indicating high similarity. Since coproporphyrin was the expected chemical entity, a siderophore of a different class (desferrioxamine B) was used to supplement the cultures in order to manipulate production of the coproporphyrin. The spectral match for coproporphyrin was found in both supplemented and unsupplemented cultures, suggesting that it is natively produced.

### ICP-MS.

Quantification of metals was accomplished using inductively coupled plasma MS (ICP-MS) of acid-digested samples. Specifically, the sample was digested in concentrated trace nitric acid (>69%; Thermo Fisher Scientific, Waltham, MA) at room temperature for 16 h. Ultrapure H_2_O (18.2 MΩ ⋅ cm) was then added to produce a final solution of 5.0% (vol/vol) nitric acid in a total sample volume of 20 ml. A quantitative standard was made using Inorganic Ventures standards IV-ICPMS-71A and IV-ICPMS-71B (Christiansburg, VA) and Fluka analytical mercury standard for ICP (Sigma-Aldrich, St. Louis, MO, USA), which were combined to create a 100-ng/ml mixed-element standard in 5.0% (vol/vol) nitric acid in a total sample volume of 50 ml.

ICP-MS was performed on a computer-controlled (QTEGRA software) Thermo iCapQ ICP-MS (Thermo Fisher Scientific, Waltham, MA) operating in standard (STD) mode and equipped with an ESI SC-2DX prepFAST autosampler (Omaha, NE). Internal standard was added inline using the prepFAST system and consisted of 1 ng/ml of a mixed-element solution containing Bi, In, ^6^Li, Sc, Tb, and Y (IV-ICPMS-71D from Inorganic Ventures). Online dilution was also carried out by the prepFAST system and used to generate calibration curves consisting of 50, 20, 10, 5, and 2 ng/ml of each element. Each sample was acquired using 1 survey run (10 sweeps) and 3 main (peak jumping) runs (40 sweeps). The isotopes selected for analysis were ^11^B; ^24,25,26^Mg; ^31^P; ^44^Ca; ^48^Ti; ^52^Cr; ^55^Mn; ^57^Fe; ^59^Co; ^60^Ni; ^63^Cu; ^66^Zn; ^77,82^Se; ^85^Rb; ^88^Sr; ^90^Zr; ^95,96^Mo; ^111^Cd; ^118^Sn; ^121^Sb; ^137^Ba; ^146^Nd; ^157^Gd; ^175^Lu; ^178^Hf; ^182^W; ^200,202^Hg; ^208^Pb; and ^6^Li, ^89^Y, ^115^In, ^159^Tb, and ^209^Bi (chosen as internal standards for data interpolation and machine stability). Instrument performance is optimized daily through autotuning followed by verification via a performance report (passing manufacturer specifications).

### RNA-seq.

To identify how growth with *Penicillium* impacts the expression of genes in the coproporphyrin biosynthesis pathway of G. arilaitensis JB182, we generated RNA-seq libraries of G. arilaitensis JB182 grown alone and G. arilaitensis JB182 grown in the presence of *Penicillium* sp. 12 (Fig. 5). A very small subset of data from this experiment focusing on expression patterns of a single operon was previously published (11). As detailed methods were previously described in that paper, we only summarize the methods here.

For each experimental unit, approximately 80,000 CFU of G. arilaitensis JB182 was spread across the surface of a 100-mm petri dish containing 20 ml of cheese curd agar. For the + *Penicillium* sp. 12 treatment, approximately 100,000 CFU of *Penicillium* was coinoculated onto the plates with G. arilaitensis JB182. Plates were kept at >90% relative humidity at 24°C for 5 days. Rind biofilms were harvested by scraping the cheese curd surface and stored in RNAprotect reagent (Qiagen), and RNA was extracted using a standard phenol-chloroform protocol as previously described (11). To deplete samples of tRNA and rRNA, both MEGAClear (Life Technologies, Inc.) and RiboZero (Illumina) kits were used. To remove both fungal and bacterial rRNA, yeast and bacterial rRNA probes from the RiboZero kits were mixed 1:2 and used for rRNA depletion. To confirm that the samples were free of DNA contaminants, a PCR of the 16S rRNA gene was performed with standard primers (27f and 1492r). Overall quantity and quality of the RNA preparations were confirmed by NanoDrop and Agilent 2100 Bioanalyzer instruments using the RNA 6000 Nano kit. RNA-seq libraries were constructed from purified mRNA using the NEBNext Ultra RNA library prep kit for Illumina (New England BioLabs). Pooled library samples were sequenced using paired-end 100-bp reads on an Illumina HiSeq Rapid Run by the Harvard Bauer Core Sequencing Core Facility. Each library yielded 6.8 to 8.9 million forward reads that were used for analysis.

To quantify gene expression and identify differentially expressed genes, we used Rockhopper (40). Only forward reads were used for this analysis. The assembled and annotated G. arilaitensis JB182 genome (NCBI GEO accession number GSE112092) was used as a reference genome for mapping. Differentially expressed genes were those deemed significantly different based on Rockhopper’s *q* values, which control for false discovery rate using the Benjamini-Hochberg procedure.

To determine the functional distributions of differentially expressed genes, amino acid sequences of genes that were 3-fold or greater differentially expressed were assigned KEGG K numbers using BlastKOALA. Membership of these genes in different KEGG pathway modules was determined using KEGG Mapper. KEGG assignments are expressed as a fraction of the genes that were differentially expressed compared to the total number of genes in the pathway.

### Growth assays with additional *Penicillium* species.

To test whether other *Penicillium* species can also cause upregulation of pink pigment production by Glutamicibacter arilaitensis JB182, 200 CFU of *Penicillium* sp. 12 and three other *Penicillium* species (P. nalgiovense, P. camemberti, and P. commune) was inoculated alone and in combination with 200 CFU of Glutamicibacter arilaitensis JB182 onto cheese curd agar in a 96-well plate. These cultures were incubated at 24°C in the dark. Photos of the interaction were taken 6 days after inoculation (see Fig. S4B in the supplemental material).

### Growth assays with purified coproporphyrin III.

To determine how purified coproporphyrin III affects the growth of G. arilaitensis JB182 and *Penicillium* sp. 12, cheese curd agar was supplemented with 0, 50, 100, and 200 µM coproporphyrin III dihydrochloride dissolved in methanol (Frontier Scientific). Cheese curd agar was distributed across 96-well plates with 150 µl of cheese curd agar per well, and wells were inoculated with 200 CFU. Each treatment was replicated four times. Ninety-six-well plates were incubated in the dark at 24°C and were sealed with a breathable film (AeraSeal; Excel Scientific, Victorville, CA). Wells were harvested at 4 and 10 days after inoculation using a sterile toothpick. CFU per well were quantified by pestling the harvested cheese curd agar plug 30 times using a micropestle in a 1.5-ml Eppendorf tube containing 600 µl of 1× phosphate-buffered saline and serially diluting the homogenate on PCAMS plates. Dunnett’s tests were used to determine changes in CFU per well between the control (0 µM) and three coproporphyrin III treatments (50, 100, and 200 µM).

### Data availability.

Raw Illumina reads and expression values have been deposited in NCBI’s Gene Expression Omnibus as GSE112092. Imaging data for analyses at pH 7 have been deposited in MassIVE under the study identifier MSV000082397 (ftp://massive.ucsd.edu/MSV000082397), and data for analyses at pH 5 are deposited under study identifier MSV000082398 (ftp://massive.ucsd.edu/MSV000082398).
