# Supplementary figures and images for: Coproporphyrin III Produced by the Bacterium Glutamicibacter arilaitensis Binds Zinc and Is Upregulated by Fungi in Cheese Rinds
Source: mSystems. 2018 Aug 21;3(4):e00036-18. doi: 10.1128/mSystems.00036-18 (PMC6104308; doi:10.1128/mSystems.00036-18)

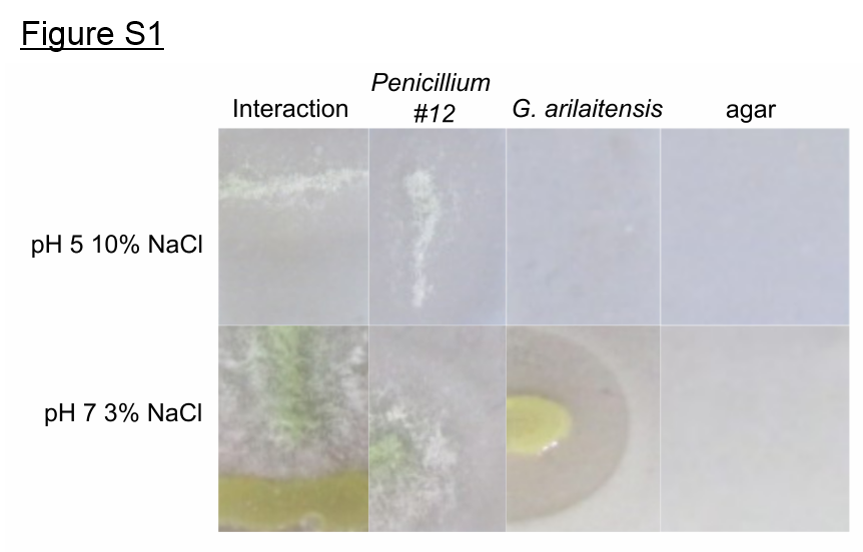

Supplement: FIG S1 [file sys004182256sf1.tif]

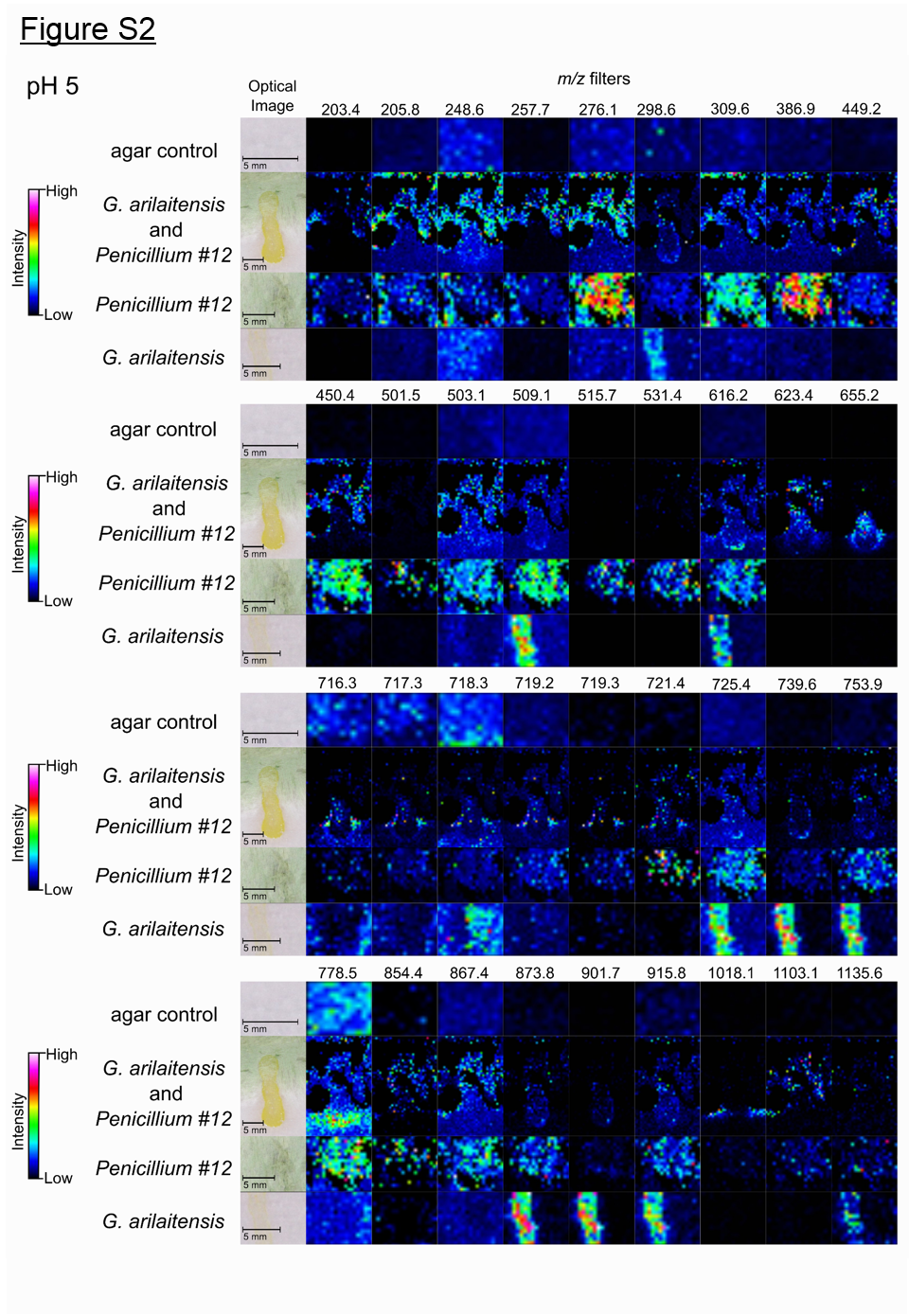

Supplement: FIG S2 [file sys004182256sf2.tif]

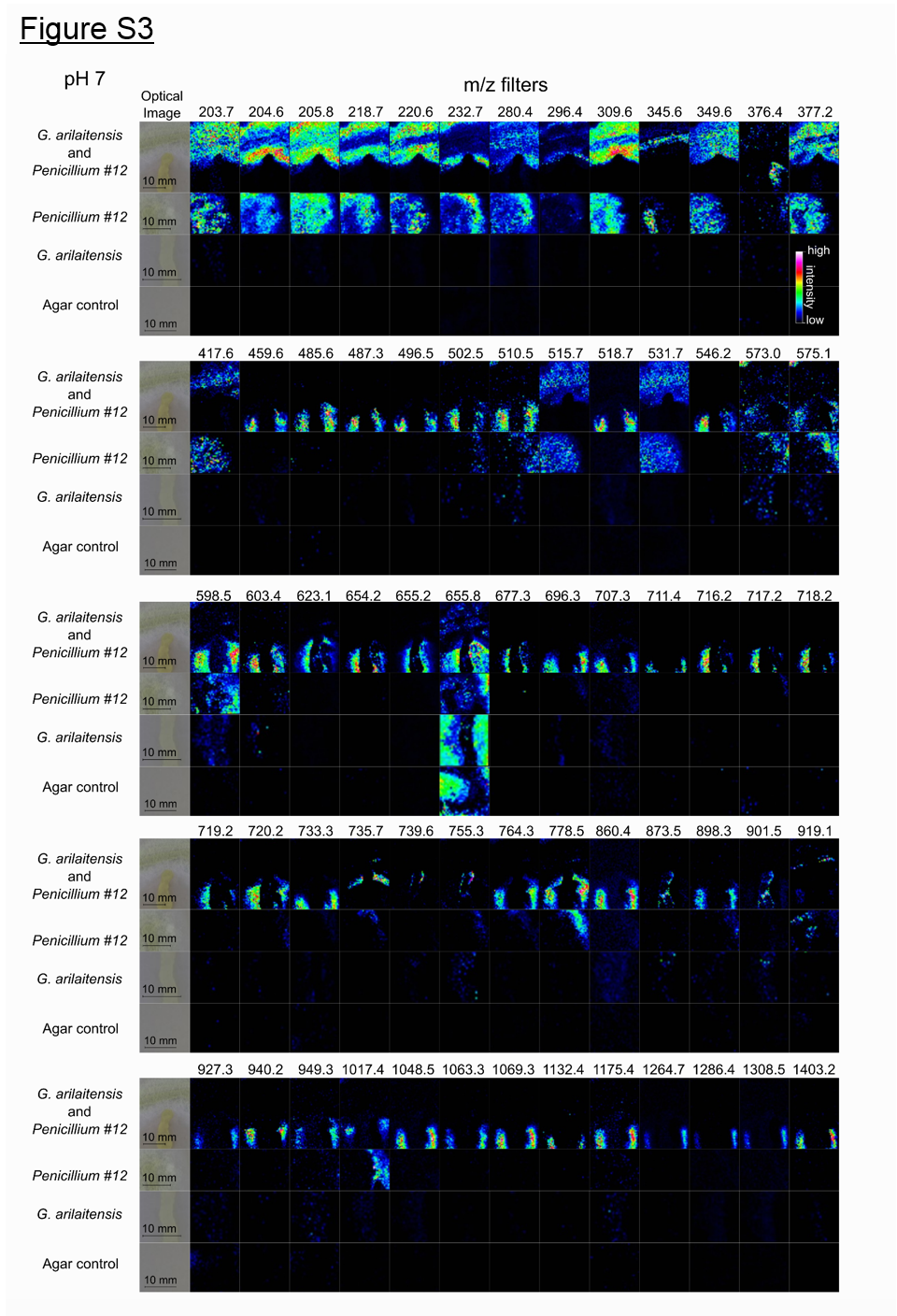

Supplement: FIG S3 [file sys004182256sf3.tif]

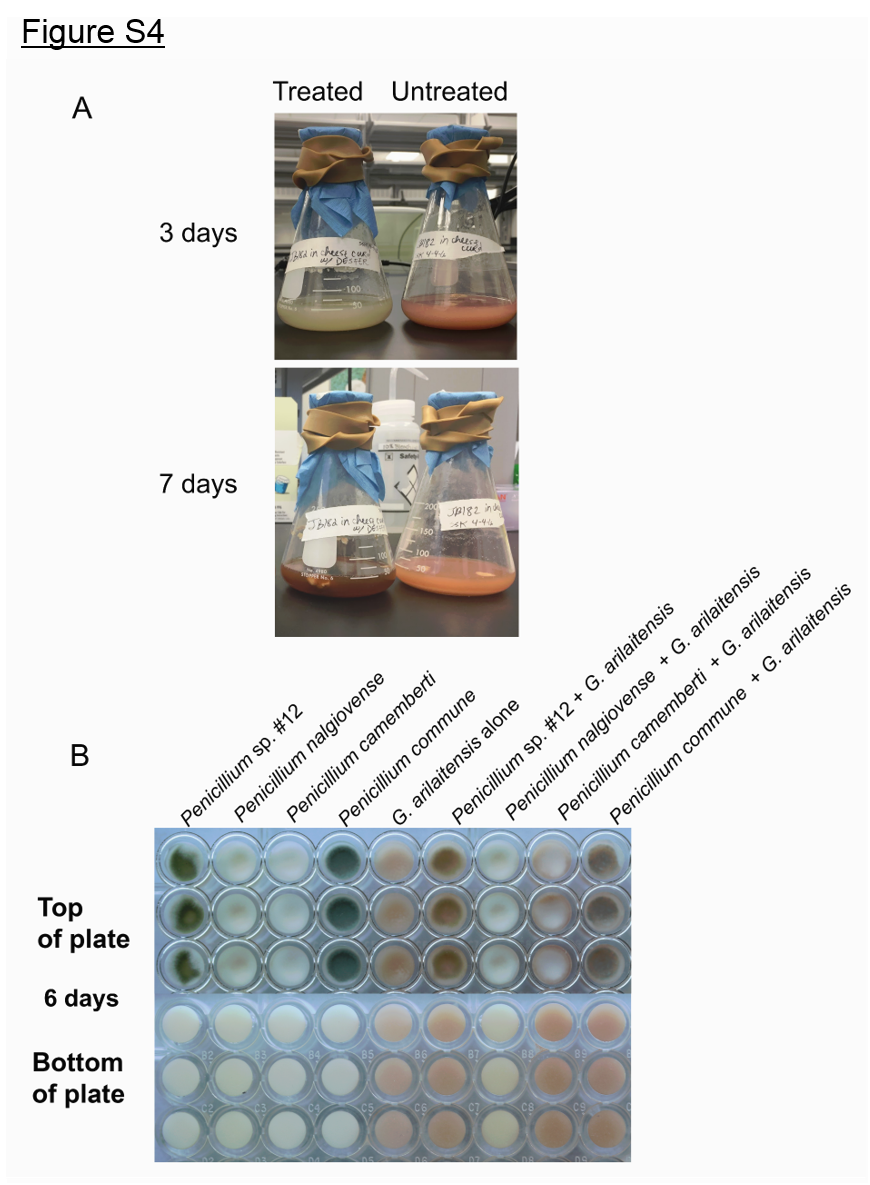

Supplement: FIG S4 [file sys004182256sf4.tif]

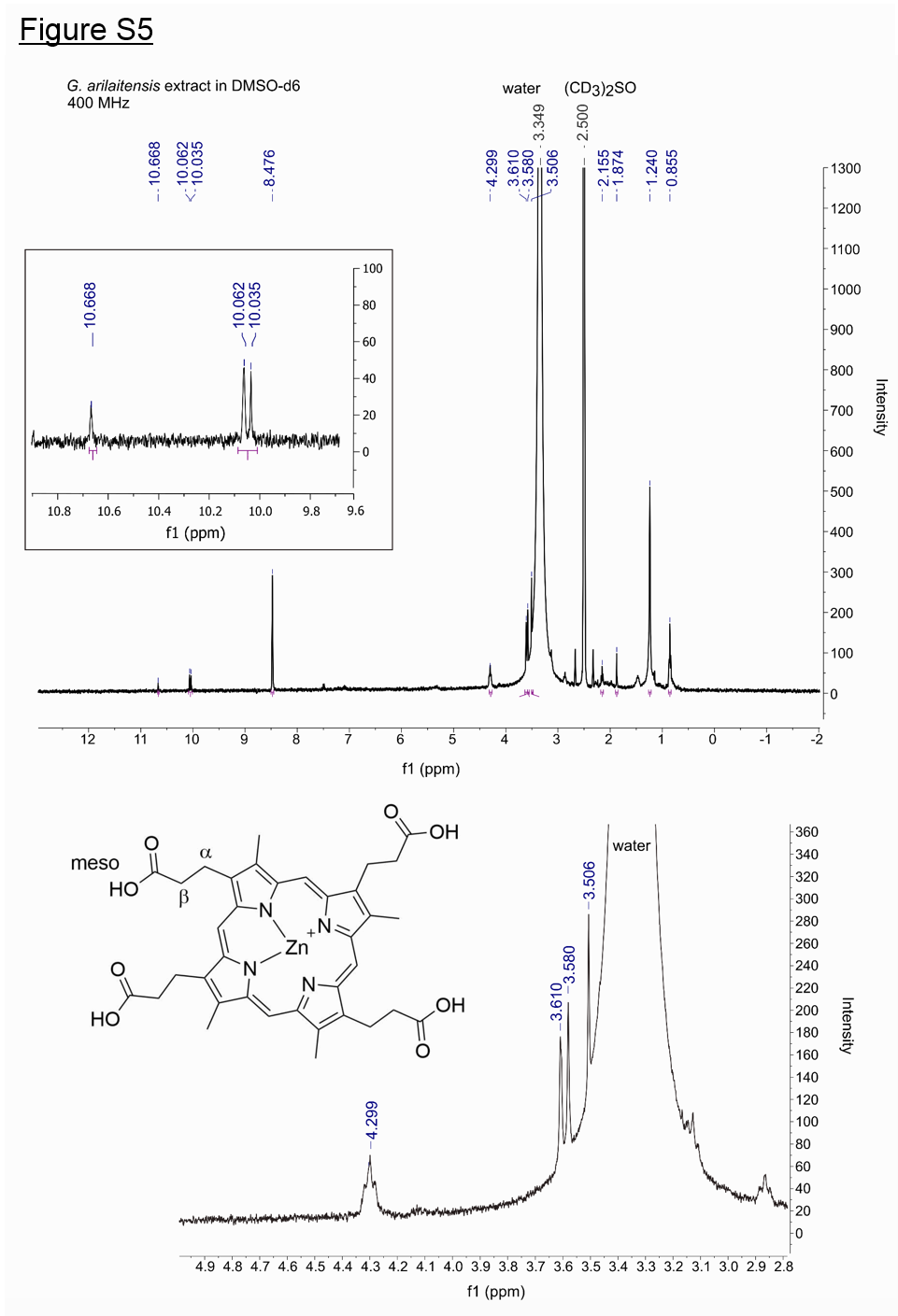

Supplement: FIG S5 [file sys004182256sf5.tif]

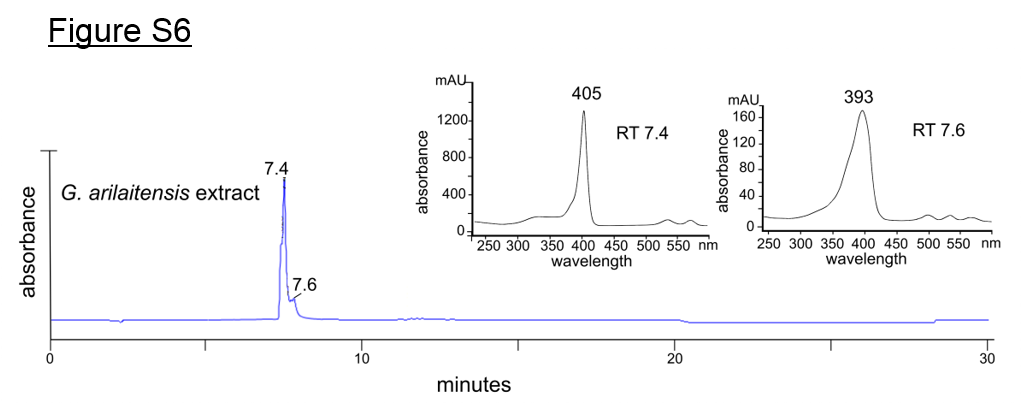

Supplement: FIG S6 [file sys004182256sf6.tif]

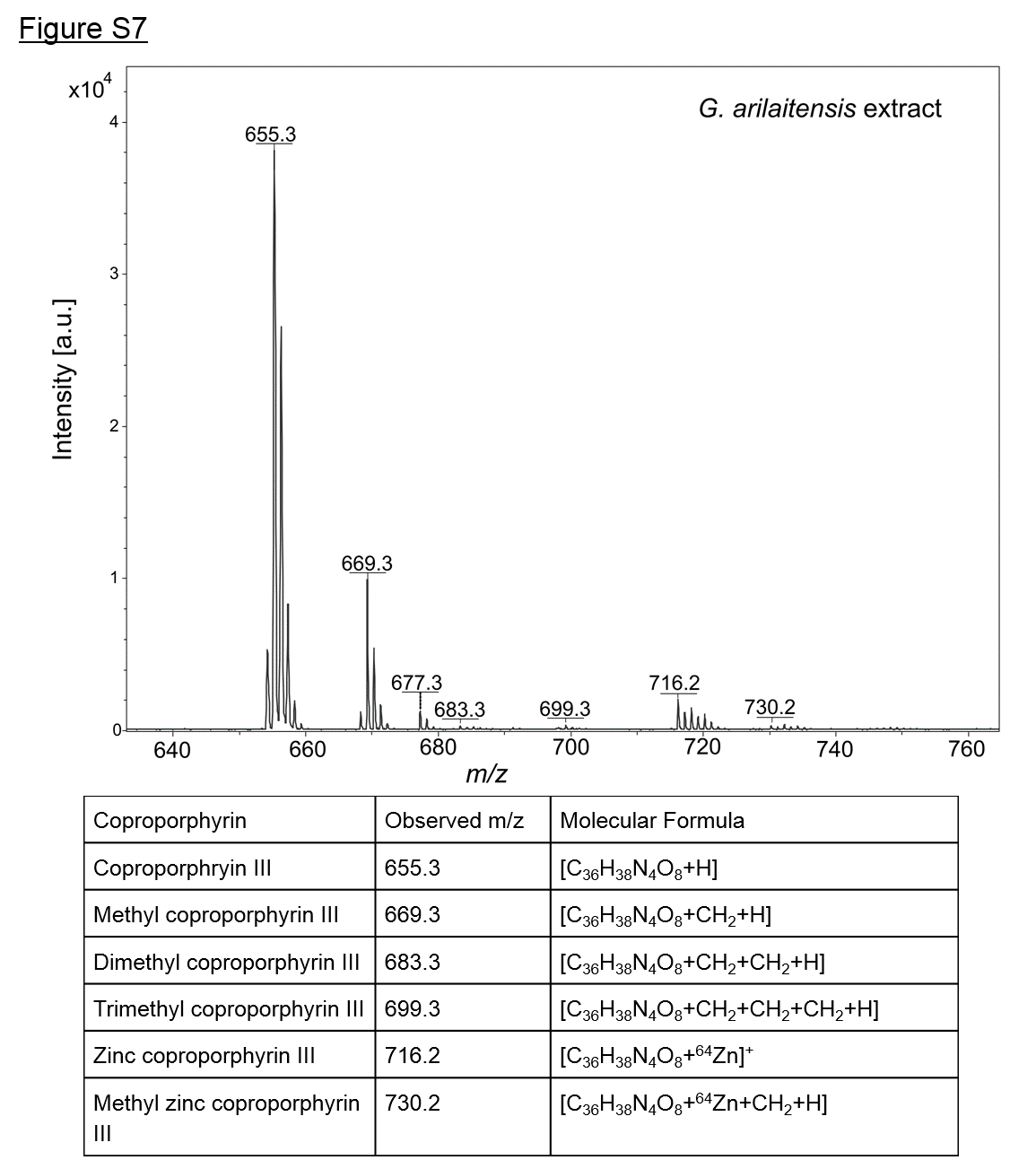

Supplement: FIG S7 [file sys004182256sf7.tif]

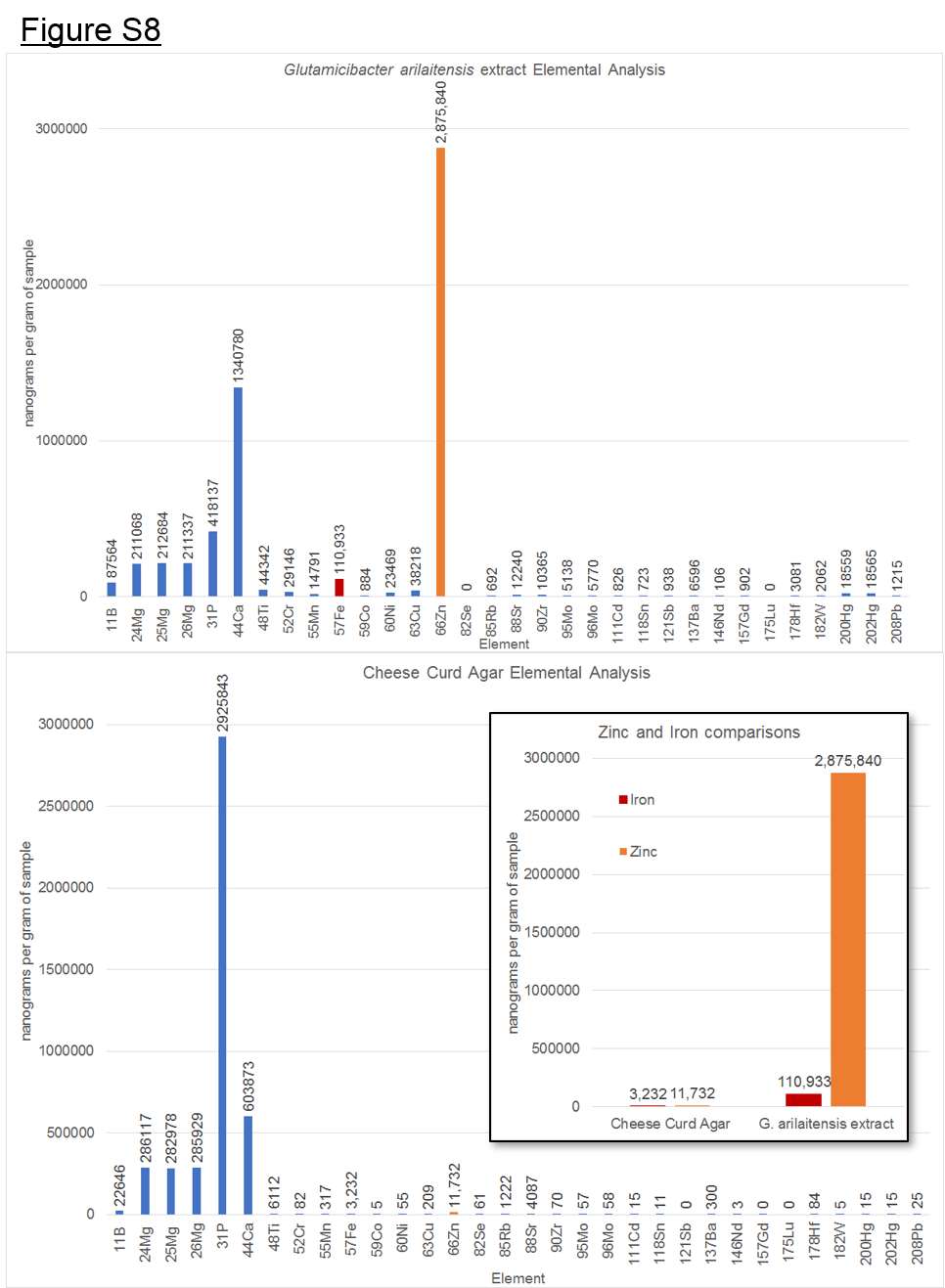

Supplement: FIG S8 [file sys004182256sf8.tif]
